# Rack ‘em, pack 'em and stack ‘em: challenges and opportunities in teaching large classes in higher education

**DOI:** 10.12688/f1000research.2-42.v1

**Published:** 2013-02-12

**Authors:** Saravana Kumar

**Affiliations:** 1 International Centre for Allied Health Evidence (iCAHE), School of Health Sciences, University of South Australia, Adelaide, 5001, Australia

## Abstract

The higher education sector is undergoing tremendous change, driven by complex driving forces including financial, administrative, and organisational and stakeholder expectations. It is in this challenging environment, educators are required to maintain and improve the quality of teaching and learning outcomes while contending with increasing class sizes. Despite mixed evidence on the effectiveness of large classes on student outcomes, large classes continue to play an important part in higher education. While large classes pose numerous challenges, they also provide opportunities for innovative solutions. This paper provides an overview of these challenges and highlights opportunities for innovative solutions.

## Commentary

There continues to be a number of significant changes to the higher education sector around the world. In Australia, these changes have been in the form of funding opportunities, student enrolments, structure and delivery of courses, and these were highlighted in the 2008 Review of Australian Higher Education ^1^. Coupled with the global financial crisis, which resulted in increasing financial pressures on higher education providers, and their funding sources, many higher education providers have taken a critical view of how courses are planned, structured and delivered. One common strategy that has been implemented is the move towards common (or foundation) courses within particular disciplines (or programs) and degrees. It is in this challenging context, educators are also required to maintain and improve the quality of teaching and learning outcomes while the student-staff ratio continues to increase ^1^.

Foundation courses are common in the first year of study at university and often encompass a number programs and degrees. As such, these courses tend to have a large cohort of students, ranging from 100 to 1500 students. These large classes pose unique challenges, including the transition from school to university and learning in a university context. While the advantages of large classes include decreased costs, improved efficiency in terms of educators’ time and resources and standardisation of learning experiences, the disadvantages include a limited range of teaching methods, limited opportunities for interaction between educators and students, and a negative perception of educators who teach large classes ^2^. While the driver for large classes may be pragmatic (such as promotion of inter-disciplinary learning) and resource-centric (such as financial), the evidence base on the effectiveness of large classes on student outcomes is mixed ^3^.

Cuseo ^4^ undertook a narrative literature synthesis of research evidence on the effect of class size on teaching, learning and retention of first year students. The findings from this literature review indicated that increasing class size had a deleterious effect on educational outcomes for students overall, and first year students in particular. Cuseo ^4^ evaluated the effect of the class size across eight different constructs including reliance on lecture method of instruction, students’ active involvement in the learning process, interaction between educators and students and feedback to students, depth of thinking inside the classroom, breadth and depth of course objectives, assignments and learning outside the classroom, students’ academic achievement and performance, students’ perspective of course satisfaction, and students’ evaluation of course instruction.

The findings from Cuseo ^4^ are supported by research undertaken by Monks and Schmidt ^5^. Drawing on data from published studies and from administrative records and student course evaluations at a private, highly selective university on the east coast of the USA, they found that both class size and student load negatively impact student assessments of courses and instructors. Large classes and heavy student loads appear to prompt faculty to alter their courses in ways deleterious to students. Other studies by ^2, 6– 8^ also highlight the potential problems associated with large classes. While there is a large body of evidence on the negative impacts of large classes, some research advice caution in interpreting these results. For example, a review of the literature by Toth and Montagna ^9^, call for future research on this topic which adequately controlled a number of variables which may influence teaching and learning outcomes. Methodological issues, such as lack of controlling of variables, definitional ambiguity on what can be considered to be important learning outcomes, the varying impact of different types of teaching methods, all contribute to the heterogeneity of the evidence base on the impact of teaching large classes.

Despite the challenges posed by large classes, due to financial and organisational drivers, large classes are going to be part of teaching and learning for most educators in the short and long term. These challenges, however, also provide opportunities for innovative thinking and strategies which may be utilised to improve the quality of teaching and learning outcomes. Burnett and Krause ^10^ provide a number of useful strategies when teaching large classes. From an organisational perspective, simple strategies such as ensuring timely and regular communication with students, providing alternate means of communication with educators (other than the traditional lecture-style didactic presentation), recognising and establishing helpful strategies on key issues students might face (such as time management, access to learning materials), and providing online and other support mechanisms might be beneficial. From a pedagogical perspective, organising and presenting effective and interesting lectures which engage the students, using multiple strategies as part of teaching (such as visual and multi-media aids, handouts, problem-based activities), encourages interaction and involvement during the teaching and learning process. This build on active learning principles, ensuring students can relate the content to real world applications and using technology to enhance learning activities.

Assessments can potentially be an important source of frustration and stress for students and educators in large classes. This can be dealt with by ensuring clear linking of course content with assessment processes, using early assessment strategies to identify potential issues, teaching students about how best to approach assessments and using automated assessments so that students can use that as a learning process.

From an affective perspective, building interest and rapport with students, taking interest in strong and weak students, ensuring there are clear pathways for communication between educators and students are important strategies to consider. Furthermore, recognising that different students learn differently using diverse means (such as visual, multimedia, and online tools) to enhance student learning opportunities and providing access to support services (such as access to teaching and learning units for international students and students from diverse backgrounds) are important as they can enhance overall student experiences.

From a management perspective, supporting and managing other educators (such as tutors) who may undertake teaching as part of the course is important to ensure consistency and standardisation in the teaching and learning process. Regular meetings among teaching staff, supporting teaching staff by providing timely access to learning materials and ensuring all the teaching staff are presented as a cohesive team are all important.

With large classes becoming entrenched in higher education, it doesn’t have to be a case of rack 'em', pack 'em' and stack ‘em’, as with challenges come opportunities. Stakeholders in education need to collaborate and partner with each other in identifying, implementing and evaluating innovative solutions. From a personal perspective, as an educator and an academic, I am tasked with coordinating a large foundation course which encompasses a number of health programs including physiotherapy, occupational therapy, medical radiations, podiatry, human movement and health sciences. Given the diversity of these programs and their respective student cohorts, my fellow educators and I face a number of challenges while delivering this course. Recognising these challenges, my fellow educators and I put in place a number of enabling strategies to address these challenges.

They include:

- Provision of clear and explicit instructions, and repeated at numerous instances, if necessary, to ensure students were fully informed about the objectives, delivery and outcomes of the course. Provision of clear and explicit instructions also extended to all educators who taught within this course to ensure consistency and uniformity in the teaching process.
- Using a range of teaching and learning approaches including lectures, tutorials, workshops, small group work, which were underpinned by active learning principles.
- A self-imposed benchmark of 24 hours response time, during working hours, to all communications from students was maintained throughout the duration of the course. This was to ensure the students’ issues were acknowledged and support provided in a timely manner.
- Regular online and face to face feedback was also provided to ensure students concerns, issues regarding the delivery and content of the course and general issues relating to the course were dealt with in a timely and efficient manner.
- The outcomes of this course were also discussed at lengths with students to ensure students were aware what this course would provide to them as future health care practitioners and how the course objectives linked with the assessment items.
- All learning materials (such as lecture and tutorial notes) were made accessible online along with podcast of lectures.
- Students were actively engaged to provide feedback about their learning experiences and this feedback was immediately implemented to ensure students could see how their feedback was utilised to inform improvements in the content and delivery of the course.
- Working in close association with the information technology staff, a number of online resources, such as discussion boards, multimedia, were created to ensure ongoing access to learning and assessment materials. The online resource was also re-constructed to make the resource user-friendly and easily accessible.
- Students who required additional assistance, such as those from non-English speaking background, international students, were provided with the opportunity to seek additional help from the educators, who, depending on the needs of the individual students, offered targeted assistance (such as referral to teaching and learning units).

While there is a growing evidence base on teaching large classes in higher education, ongoing further research is required. Future research in this area should focus on establishing the effectiveness of various models of teaching and learning and should also incorporate diverse measures of learning outcomes. With improved research evidence base, which can then inform teaching and learning practices, stakeholders in education can confidently move towards implementing evidence-informed strategies which will assist in the improvement in the quality of teaching and learning outcomes in higher education.
