# Supplementary material for: Rack ‘em, pack 'em and stack ‘em: challenges and opportunities in teaching large classes in higher education
Source: F1000Res. 2013 Feb 12;2:42. [Version 1] doi: 10.12688/f1000research.2-42.v1 (PMC3752731; doi:10.12688/f1000research.2-42.v1)
Supplement: Peer review history

# Peer review: Referee response for version 1

**Published:** 2013-02-19

**Authors:** Janet Wale

**Affiliations:** Cochrane Collaboration Consumer network, Brunswick, VIC, Australia

I agree with the author. A formal study of some of the 'interventions' used would be very interesting.

I have read this submission. I believe that I have an appropriate level of expertise to confirm that it is of an acceptable scientific standard.

# Peer review: Referee response for version 1

**Published:** 2013-02-18

**Authors:** Quinette Louw

**Affiliations:** Physiotherapy Division, University of Stellenbosch, Stellenbosch, South Africa

I have read this submission. I believe that I have an appropriate level of expertise to confirm that it is of an acceptable scientific standard.
